# Metabolic profiling of type 1 diabetes mellitus in children and adolescents: a case–control study

**DOI:** 10.1186/s13098-017-0246-9

**Published:** 2017-06-26

**Authors:** Liene Bervoets, Guy Massa, Wanda Guedens, Evelyne Louis, Jean-Paul Noben, Peter Adriaensens

**Affiliations:** 1 Faculty of Medicine and Life Sciences, Hasselt University, Martelarenlaan 42, 3500 Hasselt, Belgium; 2 Department of Pediatrics, Jessa Hospital, Stadsomvaart 11, 3500 Hasselt, Belgium; 3 Biomolecule Design Group, Applied and Analytical Chemistry, Institute for Materials Research, Hasselt University, Agoralaan 1 Building D, 3590 Diepenbeek, Belgium; 4 Biomedical Research Institute, Hasselt University, Agoralaan 1 Building C, 3590 Diepenbeek, Belgium; 5 Applied and Analytical Chemistry, Institute of Materials Research, Agoralaan 1 Building D, 3590 Diepenbeek, Belgium

**Keywords:** ^1^H-NMR spectroscopy, Metabolomics, Type 1 diabetes, Pediatrics

## Abstract

**Background:**

Type 1 diabetes mellitus (T1DM) is one of the most common pediatric diseases and its incidence is rising in many countries. Recently, it has been shown that metabolites other than glucose play an important role in insulin deficiency and the development of diabetes. The aim of our study was to look for discriminating variation in the concentrations of small-molecule metabolites in the plasma of T1DM children as compared to non-diabetic matched controls using proton nuclear magnetic resonance (^1^H-NMR)-based metabolomics.

**Methods:**

A cross-sectional study was set-up to examine the metabolic profile in fasting plasma samples from seven children with poorly controlled T1DM and seven non-diabetic controls aged 8–18 years, and matched for gender, age and BMI-SDS. The obtained plasma ^1^H-NMR spectra were rationally divided into 110 integration regions, representing the metabolic phenotype. These integration regions reflect the relative metabolite concentrations and were used as statistical variables to construct (train) a classification model in discriminating between T1DM patients and controls.

**Results:**

The total amount of variation explained by the model between the groups is 81.0% [R^2^Y(cum)] and within the groups is 75.8% [R^2^X(cum)]. The predictive ability of the model [Q^2^(cum)] obtained by cross-validation is 50.7%, indicating that the discrimination between the groups on the basis of the metabolic phenotype is valid. Besides the expected higher concentration of glucose, the relative concentrations of lipids (triglycerides, phospholipids and cholinated phospholipids) are clearly lower in the plasma of T1DM patients as compared to controls. Also the concentrations of the amino acids serine, tryptophan and cysteine are slightly decreased.

**Conclusions:**

The present study demonstrates that metabolic profiling of plasma by ^1^H-NMR spectroscopy allows to discriminate between T1DM patients and controls. The metabolites that significantly differ between both groups might point to disturbances in biochemical pathways including (1) choline deficiency, (2) increased gluconeogenesis, and (3) glomerular hyperfiltration. Although the sample size of this study is still somewhat limited and a validation should be performed, the proof of principle looks promising and justifies a deeper investigation of the diagnostic possibilities of ^1^H-NMR metabolomics in follow-up studies.

*Trial registration* NCT03014908. Registered 06/01/2017. Retrospectively registered

**Electronic supplementary material:**

The online version of this article (doi:10.1186/s13098-017-0246-9) contains supplementary material, which is available to authorized users.

## Background

Type 1 diabetes mellitus (T1DM) is one of the most common pediatric diseases and its incidence is rising in many countries [1]. T1DM is a chronic metabolic disorder that results from a lack of pancreatic β-cell insulin production by autoimmune mechanisms [2]. Insulin is a key hormone to maintain metabolic homeostasis, regulating carbohydrate, lipid and protein metabolism, and insulin deficiency in T1DM subsequently induces a variety of metabolic derangements [3, 4]. To identify novel pathways or early biomarkers indicative of metabolic alterations that are involved in the development of diabetes, metabolomics is an increasingly used tool [5]. Metabolomics research on pediatric study populations is still in its infancy. Up to now, only a few researchers investigated the plasma metabolic fingerprint of T1DM in children, using mass spectrometry as analytical tool [6–8]. One study showed that children who later progressed to T1DM had reduced serum levels of succinic acid and phosphatidylcholine at birth, pointing towards a dysregulated metabolism preceding β-cell autoimmunity and overt T1DM [6]. In addition, methionine deficits in early childhood may lead to an increased risk to develop T1DM later in life [7]. When comparing the plasma metabolic profile of T1DM and healthy children, differences were observed in lipid metabolism (non-esterified fatty acids, lysophospholipids and other fatty acid-derivatives), and some markers of differential activity of the gut microbiota (bile acids, p-cresol sulfate) [8]. However, the use of nuclear magnetic resonance (NMR)-based metabolomics to obtain a deeper knowledge of the plasma metabolic profile of T1DM has not been fully explored in the pediatric population. Proton (^1^H)-NMR spectroscopy has proven to be a robust and reproducible technique with very limited sample preparation (no extractions) [9], that can detect and quantify a wide variety of metabolites simultaneously, providing information regarding the biochemical pathways involved [10]. The objective of the current study was to investigate metabolic variations in the plasma of T1DM children and adolescents as compared to plasma of non-diabetic matched controls using ^1^H-NMR spectroscopy combined with multivariate statistics.

## Methods

### Subjects and characteristics

Children with poorly controlled T1DM (n = 7) and non-diabetic controls (n = 7) were recruited at the Department of Pediatrics of the Jessa Hospital Hasselt (Belgium) between June 2012 and November 2013. Inclusion criteria were: (1) aged between 8 and 18; (2) normal-weight according to the International Obesity Task Force (IOTF) BMI criteria [11]; and (3) fasted for at least 8 h. All subjects were matched for gender (four males and three females in both groups), age (12.0 ± 3.0 and 13.5 ± 2.7 years, respectively), and BMI-SDS (0.03 ± 0.62 and 0.05 ± 0.55, respectively). Subject characteristics are presented in Table 1. T1DM patients were diagnosed according to international consensus guidelines [12]. T1DM patients had diabetes for 6.8 ± 3.8 years, and were treated with exogenous insulin (mean insulin dose per kg: 0.83 ± 0.22/kg). T1DM patients show high fasting plasma glucose levels (mean: 187 ± 82 mg/dl) and hemoglobin A1c (HbA1c) concentrations above 6.5% (mean: 9.7 ± 2.8%) (Additional file 1: Table S1). None of the subjects was using lipid-lowering drugs or other medication. The study was conducted in accordance with the ethical rules of the Helsinki Declaration and Good Clinical Practice. The study protocol was approved by the medical-ethical committees of the Jessa Hospital and Hasselt University (12.27/ped12.02). Informed and written consent was obtained from all participants and their parents or legal guardian.

**Table 1:** Subject characteristics

Subject alias	Age	Gender	Tanner stage	Height (in cm)	Weight (in kg)	BMI	BMI-SDS
T1DM patients							
1	17.2	1	5	167.0	54.0	19.4	−0.38
2	15.0	0	5	159.0	58.0	22.9	1.01
3	11.4	1	2	152.0	37.0	16.0	−0.52
4	10.0	0	1	148.0	34.5	15.8	−0.31
5	8.7	0	1	140.0	33.2	16.9	0.59
6	11.8	1	2	149.0	40.5	18.2	0.37
7	10.1	1	1	139.5	30.0	15.4	−0.53
Controls							
1	15.0	1	5	172.8	58.9	19.7	0.23
2	9.9	1	1	139.0	30.3	15.7	−0.38
3	11.6	1	1	147.0	42.2	19.5	0.92
4	12.6	0	2	158.7	40.9	16.2	−0.81
5	12.5	0	3	151.9	41.0	17.8	−0.11
6	17.8	0	5	168.4	60.1	21.2	0.14
7	15.2	1	4	179.1	64.1	20.0	0.32

No significant differences were found between T1DM and control group for age, gender, Tanner stage (pubertal status), height, weight, BMI and BMI-SDS

*T1DM* type 1 diabetes, *BMI* body mass index, *BMI-SDS* body mass index standard deviation score

### Biochemical measurements

Fasting venous blood of T1DM patients was collected in 2-ml fluoride-oxalate tubes for biochemical analysis at the Clinical Laboratory of Jessa Hospital. Plasma glucose was measured by the glucose oxidase method using a Synchron LX20 analyzer (Beckman Coulter, Brea, CA, USA) and HbA1c was measured using ion exchange chromatography (Menarini HA-8160 HbA1c auto-analyzer, Menarini Diagnostics, Belgium).

### Sample collection, preparation and ^1^H-NMR analysis

Fasting venous blood was collected in 6-ml lithium heparin tubes and stored at 4 °C within 10 min. Within 30 min, samples were centrifuged at 1600*g* for 15 min and plasma aliquots of 500 µl were transferred into cryovials and stored at −80 °C [13]. Detailed protocols regarding sample preparation and ^1^H-NMR analysis have been previously described elsewhere [14]. Plasma ^1^H-NMR spectra were rationally divided into 110 integration regions defined on the basis of spiking experiments with known metabolites [15]. These integration regions reflect the relative metabolite concentrations—i.e. the metabolic phenotype—and were used as statistical variables to construct (train) a classification model in discriminating between T1DM patients and controls.

### Statistical analysis

Multivariate statistics was performed using SIMCA-P^+^ (Version 13.0, Umetrics, Sweden). After mean-centering and Pareto scaling of the variables, unsupervised principal component analysis (PCA) was performed in order to look for clustering and possible confounders within the dataset, and to identify possible outliers by a Hotelling’s T2 range test and a distance to model plot. In a next step, orthogonal partial least squares discriminant analysis (OPLS-DA) was used to build (train) a model (statistical classifier) to discriminate between T1DM patients and controls [16]. The validity of the established model was evaluated on one hand by the total amount of variation between and within the groups explained by the model [denoted as R^2^Y(cum) and R^2^X(cum), respectively] and on the other hand by the predictive ability of the model as determined by a sevenfold cross-validation [denoted as Q^2^(cum)]. To be classified as a variable that strongly contributes to the group discrimination, three selection criteria have to be fulfilled: (1) significantly different in univariate statistics (a student t test corrected for multiple testing by the Benjamini–Hochberg method), (2) an OPLS-DA absolute value of p(corr), i.e. the loading scaled as a correlation coefficient, exceeding 0.6 and (3) an OPLS-DA variable importance for the projection (VIP) value exceeding 0.5 [16].

## Results

**Fig. 1:**
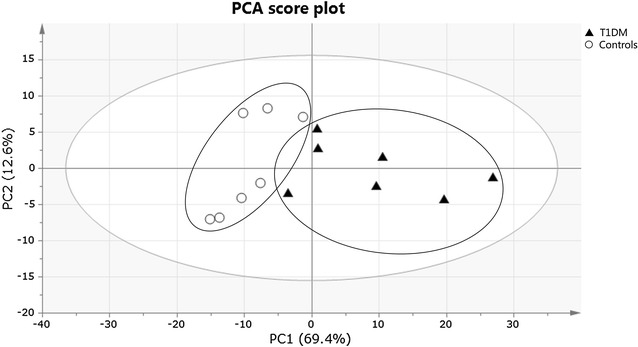
PCA score plot obtained for T1DM patients (*filled triangle*) and healthy controls (*circle*). Each participant is represented by its metabolic profile and visualized as a single symbol of which the location is determined by the contributions of the 110 variables in the ^1^H-NMR spectrum. The PCA score plot shows the first principal component (PC1: 69.4%), explaining the largest variance within the dataset, versus the second principal component (PC2: 12.6%) that explains the second largest variance

Multivariate OPLS-DA statistics was used to train a classification model (classifier) in discriminating between T1DM patients and controls based on data input from their metabolic profile or phenotype. A PCA analysis was conducted first to look for clustering and possible confounders. Figure 1 shows that the subjects were clustered in a way that allowed T1DM patients to be clearly differentiated from controls and no outliers were detected. Moreover, staining the PCA score plots for gender, age and BMI-SDS clearly shows that none of these factors have a confounding effect on the discrimination between T1DM patients and controls, as was expected for matched groups (data not shown).

**Fig. 2:**
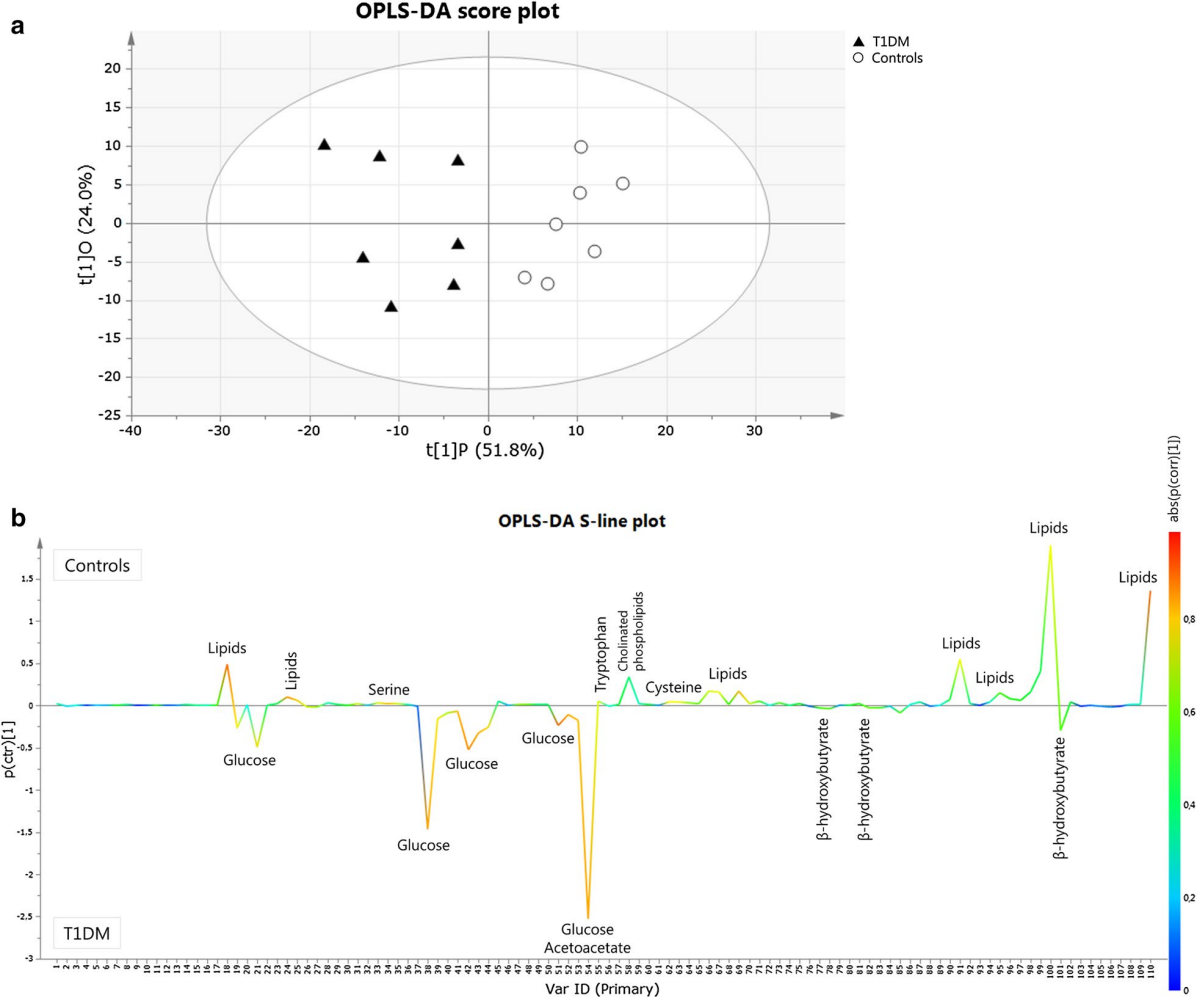
OPLS-DA score plot (**a**) and S-line plot (**b**) obtained for T1DM patients (*filled triangle*) and healthy controls (*circle*). Each participant is represented by its metabolic profile and visualized as a single symbol of which the location is determined by the contributions of the 110 variables in the ^1^H-NMR spectrum. The OPLS-DA score plot shows the first predictive component (t[1]P: 51.8%), explaining the variation between the groups, versus the first orthogonal component (t[1]O: 24.0%) that explains the variation within the groups. The OPLS-DA S-line plot visualizes differences between T1DM patients (negative) and controls (positive). The left *y-axis* represents p(ctr)[1], the covariance between a variable and the classification score. It indicates if an increase or decrease of a variable is correlated to the classification score. The magnitude of the covariance is however difficult to interpret since covariance is scale dependent. This means that a high value for the covariance does not necessary imply a strong correlation, as the covariance is also influenced by the intensity of the signal with respect to the noise level. Therefore this measure will likely indicate variables with large signal intensities. The right *y-axis* shows p(corr)[1], the correlation coefficient between a variable and the classification score (i.e. the normalized covariance). It gives a linear indication of the strength of the correlation. As the correlation is independent of the intensity of the variable, it will be a better measure for the reliability of the variable in the classification process. In  **b**, the *red color* stands for the highest absolute value of the correlation coefficient. Strongly discriminating variables have a large intensity and large reliability

In a next step, OPLS-DA was used to build a model (statistical classifier) to differentiate between T1DM patients and controls (Fig. 2a). The total amount of variation between the groups that can be explained by the model is 81.0% [R^2^Y(cum)] while this within the groups is 75.8% [R^2^X(cum)]. The predictive ability of the model, obtained by cross-validation, is quite high with a Q^2^(cum) of 50.7%, indicating that the discrimination between the groups on the basis of the metabolic phenotype is valid. The OPLS-DA S-line plot shown in Fig. 2b visualizes the covariance [left y-axis; p(ctr)] and correlation coefficient [right y-axis; abs(p(corr)] between the variables and the classification score in the model (see caption of Fig. 2b for more information). Strongly discriminating variables combine a clear covariance [p(ctr)] with a high absolute value for the correlation coefficient [abs(p(corr)] (Table 2).

**Table 2:** Plasma variables that significantly differ between T1DM and control subjects by multivariate statistics

VAR	Spectral range (ppm)	Assigned metabolite	Model based on all 110 VARs (including glucose related VARs)		Model based on 95 VARs (excluding glucose related VARs)	
			p(corr)[1]	VIP ± cvSE	p(corr)[1]	VIP ± cvSE
18	5.4300–5.2752	–**H**C=C**H**– in fatty acid chain	0.890	2.07 ± 0.71	0.787	2.77 ± 0.82
24	4.4100–4.3159	C_1_ **H** and C_3_ **H** in glycerol backbone of triglycerides + OC**H**_2_ of choline in sphingomyelin/phosphatidylcholine	0.856	0.89 ± 0.47	0.740	1.19 ± 0.63
34	4.0310–4.0136	l-Serine	0.809	0.48 ± 0.30	0.743	0.65 ± 0.40
35	4.0136–4.0010	l-Serine	0.715	0.43 ± 0.26	0.669	0.57 ± 0.38
38	3.9590–3.8330	d-Glucose	−0.837	3.56 ± 0.24		
41	3.7956–3.7820	d-Glucose	−0.815	0.83 ± 0.24		
42	3.7820–3.7550	d-Glucose	−0.861	2.19 ± 0.12		
43	3.7550–3.7390	d-Glucose	−0.825	1.66 ± 0.14		
44	3.7390–3.7141	d-Glucose	−0.782	1.42 ± 0.19		
51	3.5914–3.5649	d-Glucose	−0.901	1.44 ± 0.27		
52	3.5649–3.5510	d-Glucose	−0.828	1.04 ± 0.19		
54	3.5360–3.3980	d-Glucose	−0.836	4.63 ± 0.54		
55	3.3980–3.3765	l-Tryptophan	0.721	0.81 ± 0.28	0.870	1.08 ± 0.35
63	3.1090–3.0860	l-Cysteine	0.754	0.57 ± 0.37	0.701	0.77 ± 0.54
91	2.1230–1.9720	–C**H**_2_–CH=CH– in fatty acid chain	0.752	2.17 ± 0.44	0.734	2.91 ± 0.65
100	1.3450–1.2458	CH_3_–(C**H**_2_)_n_– in fatty acid chain	0.747	4.04 ± 1.52	0.728	5.41 ± 1.75
110	0.9660–0.8000	C**H**_3_–(CH_2_)_n_– in fatty acid chain	0.898	3.14 ± 0.73	0.735	4.21 ± 1.03

*cvSE* standard error of cross-validation, *p(corr)[1]* correlation scaled loading, *VAR* variable, *VIP* variable influence on projection

The plot shows that the concentrations of lipids (triglycerides, phospholipids and cholinated phospholipids) are clearly decreased in the plasma of T1DM patients as compared to controls, whereas serine, tryptophan and cysteine concentrations seem to be decreased slightly. The glucose levels on the other hand are clearly increased in the plasma of T1DM patients. These changes were also observed as significant by a univariate *t* test with post hoc Benjamini–Hochberg correction (Table 3). The plot further shows that ketone levels (i.e. acetoacetate and β-hydroxybutyrate) are slightly elevated in T1DM patients. In order to look if T1DM patients and controls can be differentiated by a model constructed without the 15 variables related to the strong glucose signals in the ^1^H-NMR spectra, the variables representing glucose were removed from the metabolic profile prior to the OPLS-DA model building (construction of the model with only 95 variables). The results are also presented in Table 2 and confirm that the relative concentrations of lipids (triglycerides, phospholipids and cholinated phospholipids) and some amino acids (serine, tryptophan and cysteine) are reduced in the plasma of T1DM patients.

**Table 3:** Plasma variables that significantly differ between T1DM and control subjects by univariate statistics

VAR	Spectral range (ppm)	Assigned metabolite	Relative concentration		Increased/decreased in T1DM	p value*	%-change
			T1DM	Controls			
18	5.4300–5.2752	–**H**C=C**H**– in fatty acid chain	21.95 ± 3.63	30.95 ± 4.00	↓	0.001	−29.1
24	4.4100–4.3159	C_1_ **H** and C_3_ **H** in glycerol backbone of triglycerides + OC**H**_2_ of choline in sphingomyelin/phosphatidylcholine	3.28 ± 1.16	5.09 ± 1.16	↓	0.005	−35.6
34	4.0310–4.0136	l-Serine	0.70 ± 0.26	1.21 ± 0.22	↓	0.003	−42.1
35	4.0136–4.0010	l-Serine	0.84 ± 0.28	1.29 ± 0.23	↓	0.008	−34.9
38	3.9590–3.8330	d-Glucose	91.99 ± 16.9	64.44 ± 6.58	↑	0.004	42.8
41	3.7956–3.7820	d-Glucose	7.62 ± 0.60	6.18 ± 0.60	↑	<0.001	23.3
42	3.7820–3.7550	d-Glucose	30.58 ± 5.63	20.56 ± 1.95	↑	0.003	48.7
43	3.7550–3.7390	d-Glucose	17.92 ± 3.83	11.85 ± 1.35	↑	0.005	51.2
44	3.7390–3.7141	d-Glucose	17.76 ± 3.24	13.05 ± 1.49	↑	0.007	36.1
51	3.5914–3.5649	d-Glucose	14.45 ± 2.19	10.20 ± 1.06	↑	0.002	41.7
52	3.5649–3.5510	d-Glucose	5.84 ± 1.10	3.65 ± 0.50	↑	0.002	60.0
54	3.5360–3.3980	d-Glucose	116.37 ± 30.40	69.27 ± 8.54	↑	0.005	68.0
55	3.3980–3.3765	l-Tryptophan	0.70 ± 0.32	1.94 ± 0.41	↓	<0.001	−63.9
63	3.1090–3.0860	l-Cysteine	2.28 ± 0.47	3.05 ± 0.39	↓	0.006	−25.2
91	2.1230–1.9720	–C**H**_2_–CH=CH– in fatty acid chain	53.13 ± 7.19	64.03 ± 3.76	↓	0.007	−17.0
100	1.3450–1.2458	CH_3_–(C**H**_2_)_n_– in fatty acid chain	117.69 ± 17.62	155.40 ± 22.01	↓	0.004	−24.3
110	0.9660–0.8000	C**H**_3_–(CH_2_)_n_– in fatty acid chain	89.10 ± 14.79	111.75 ± 7.83	↓	0.006	−20.3

* Benjamini–Hochberg adjusted p value, calculated using the independent samples *t* test. %-change is the increase (+) or decrease (−) of the mean in the T1DM group with respect to the control group

*VAR* variable

## Discussion

Type 1 diabetes mellitus is a serious health concern worldwide that is usually diagnosed in children and young adults [1]. T1DM is a metabolic disorder, and in recent decades it has been shown that metabolites other than glucose play an important role in insulin deficiency and the development of diabetes [4, 5]. Metabolomics, the study of small-molecule metabolites, has developed into an important tool in diabetes research [5]. In this study, we investigated metabolic variations in T1DM children and adolescents using NMR-spectroscopy-based metabolomics to gain inside into biochemical pathways that are altered in early stages of T1DM. Besides the expected higher concentration of glucose, we found lower relative concentrations for lipids (triglycerides, phospholipids and cholinated phospholipids) and the amino acids serine, tryptophan and cysteine in plasma of T1DM children and adolescents as compared to non-diabetic controls.

Our findings of relatively lower levels of lipids in the plasma of T1DM as compared to controls are in agreement with other metabolomics studies [3, 4, 6]. In a prospective Finnish study, it was found that children who developed T1DM have reduced serum levels of phosphatidylcholine at birth, next to lower levels of multiple triglycerides and phospholipids throughout the follow-up [6]. In addition, it has been demonstrated that children developing T1DM before 4 years of age have low cord-blood levels of phospholipids, mainly phosphatidylcholines [17]. It is suggested that T1DM progressors are choline-deficient at birth, and that choline deficiency is the key mechanism leading to lower serum triglyceride levels and their increased accumulation in the liver [6]. Choline metabolism also depends on the gut microbial composition [18], making the latter an attractive target for early prevention and treatment of T1DM [19]. In addition, low levels of phosphatidylcholine may play a role in oxidative damage affecting the pancreatic β-cell insulin production, because phosphatidylcholines are thought to have anti-inflammatory properties [20]. According to an NMR-based metabolomics study in adults, lower levels of triglycerides in T1DM patients may also be attributable to treatment with insulin [3], which is known to have an anti-lipolytic action [21]. We further observed lower plasma levels of serine, tryptophan and cysteine in T1DM as compared to controls. In current literature, only a limited number of papers can be found regarding the relationship between these amino acids and T1DM. A study in diabetic db−/db− mice suggested that a strongly decreased concentration of the gluconeogenic amino acids serine, alanine and glycine, resulted in increased gluconeogenesis [22]. In addition, a study in rats suggested that tryptophan suppresses the elevation of blood glucose and lessens the burden associated with insulin secretion from β-cells [23]. Finally, reduced plasma levels of cysteine in T1DM patients can be explained by glomerular hyperfiltration, resulting in an increased renal clearance of cysteine [24].

Although the sample size of this study is still somewhat limited, the experiments were carried out according to a strictly controlled protocol. This pilot study demonstrates the proof of principle that metabolic phenotyping of T1DM in children by proton-NMR spectroscopy allows to differentiate between T1DM patients and controls and therefore justifies the start-up of larger studies.

Because NMR metabolomics can be used to search for subtle changes in the plasma of children prone to develop T1DM, it might become an important tool for the early diagnosis and prognosis of T1DM in children. Hence, restoring or improving the plasma metabolic profile, e.g. by re-establishing lipid and amino acid availability or by modulating gut microbial composition, might prevent β-cell destruction and delay T1DM progression in children and adolescents.

## Conclusion

The present study demonstrates the proof of principle that metabolic phenotyping of plasma by ^1^H-NMR spectroscopy allows to discriminate between T1DM patients and controls. T1DM children and adolescents show lower relative plasma concentrations of lipids (triglycerides, phospholipids and cholinated phospholipids), serine, tryptophan and cysteine as compared to non-diabetic controls. NMR-spectroscopy-based metabolomics appears to be a promising tool for the identification of disturbed biochemical pathways related to the development of T1DM. Nevertheless, further identification and validation is needed in order to evaluate the use of NMR metabolomics in the prediction, diagnosis and monitoring of T1DM in children. Therefore, deeper follow-up studies in larger pediatric cohorts are of utmost importance to further explore the potential of metabolomics in the field of pediatric diabetes.

## Additional file

**Additional file 1: Table S1.** Characteristics of T1DM patients.

## Abbreviations

^1^H-NMR: proton nuclear magnetic resonance
BMI: body mass index
BMI-SDS: body mass index standard deviation score
cvSE: standard error of cross-validation
HbA1c: hemoglobin A1c
IOTF: International Obesity Task Force
OPLS-DA: orthogonal partial least squares discriminant analysis
PCA: principal component analysis
p(corr)[1]: correlation scaled loading
T1DM: type 1 diabetes mellitus
VAR: variable
VIP: variable influence on projection
