# Supplementary material for: Metabolic profiling of type 1 diabetes mellitus in children and adolescents: a case–control study
Source: Diabetol Metab Syndr. 2017 Jun 26;9:48. doi: 10.1186/s13098-017-0246-9 (PMC5485735; doi:10.1186/s13098-017-0246-9)
Supplement: Supplementary file 1 — Additional file 1: Table S1. Characteristics of T1DM patients. [file 13098_2017_246_MOESM1_ESM.docx]

**Table S1 Characteristics of T1DM patients**

| **Subject alias T1DM patients** | **Number of years having T1DM** | **Insulin dose per kg** | **HbA1c (%)** | **HbA1c (mmol/mol)** | **Fasting glucose (mg/dl)** | **Total cholesterol (mg/dl)** | **HDL-C (mg/dl)** | **LDL-C (mg/dl)** | **Triglycerides**  **(mg/dl)** | **AST (U/l)** | **ALT (U/l)** | **γ –GT**  **(U/l)** |
| --- | --- | --- | --- | --- | --- | --- | --- | --- | --- | --- | --- | --- |
| 1 | 14,7 | 1,30 | 15,9 | 150 | 104 | 156 | 40 | 102 | 68 | 17 | 14 | 13 |
| 2 | 5,3 | 0,67 | 7,7 | 61 | 292 | 231 | 60 | 151 | 98 | 30 | 27 | 14 |
| 3 | 4,1 | 0,78 | 8,4 | 68 | 121 | 192 | 91 | 93 | 42 | 29 | 21 | 15 |
| 4 | 6,0 | 0,84 | 9,8 | 84 | 165 | 135 | 57 | 65 | 65 | 33 | 17 | 16 |
| 5 | 3,7 | 0,65 | 9,2 | 77 | 292 | 125 | 49 | 64 | 60 | 23 | 16 | 16 |
| 6 | 5,4 | 0,85 | 8,8 | 73 | 114 | 152 | 57 | 81 | 68 | 24 | 16 | 16 |
| 7 | 8,7 | 0,72 | 8,4 | 68 | 218 | 178 | 73 | 93 | 60 | 30 | 22 | 14 |

ALT: alanine transaminase; AST: aspartate aminotransferase; γ-GT: gamma-glutamyltransferase; HbA1c: hemoglobin A1c; HDL-C: high-density lipoprotein cholesterol; LDL-C: low-density lipoprotein cholesterol.
